# A Deficiency in the Hospital Service

**Published:** 1912-11-02

**Authors:** 


### A DEFICIENCY IN THE HOSPITAL SERVICE.

BY A PROVINCIAL HOSPITAL SECRETARY.

Whilst it is quite impossible to forecast the future of the Voluntary Hospital Service, owing to the fact that the period through which we are passing is so fruitful of legislation, the future will be largely influenced by the ability of hospital managers to maintain their institutions. Inasmuch as the preservation of the Voluntary Service is the earnest desire of all those who fully appreciate the advantages that such a Service possesses over a State or Municipal Service, it is desirable that hospital managers should consider as to what means may be adopted not only to maintain, but to improve the present standard of efficiency.

A high standard of efficiency is as necessary in the interest of financial prosperity as it is for the physical welfare of the community, but we must admit that there is a wide inequality in the financial prosperity of the voluntary hospitals of this country, and it does not invariably follow, as it should do, that the areas most in need of a good Service are so provided; indeed, the reverse is sometimes the case.

It may, perhaps, be assumed that the prosperity of a hospital is dependent upon the abilities and activities of its managers. To a large extent this may be the case, but there are many other determining factors, and not the least amongst the most important of these is one which arises from the facts that many of our hospitals are not in themselves complete units of the Service, and that there is no system of co-operation amongst the hospitals whereby the required complement may be supplied at other institutions. Many of the smaller hospitals, owing to the lack of the more expensive appliances, of special departments, and of the services of consultants or specialists, are not in a position to deal adequately with some of the most serious cases, yet there is no methodical process whereby such cases which present themselves for treatment can be drafted to those institutions specially equipped for dealing with them. The patients may ultimately drift, individually, to suitable hospitals, but meanwhile valuable time is lost and the relief of the patient prejudiced. Our hospitals should at least be in the position to do as well for their patients as is the private practitioner for his, who, when the need is indicated, seeks the services of the consultant. It must be within the experience of many hospital managers that, owing to the lack of the system of co-operation referred to, patients are at times admitted to their hospitals, who, by reason of the lengthy period of treatment the nature of their illnesses demands, can only be detained until they are tided over the acute stage of their illnesses. Cases of this description sometimes occupy beds which could be made use of to greater advantage, not because of imperfect supervision of admissions, but because it is urgently necessary to do something for their relief, and no other means are at hand. This lack of co-operation seems to be a serious defect in the Voluntary Service which cannot but react adversely upon its financial prosperity, for in the result many of our hospitals lose prestige and a corresponding proportion of support, and, on the other hand, the institutions which give the desired relief upon the individual applications of the patients do not benefit to the extent of this loss.

The British Hospitals Association is doing a great deal toward promoting co-operation amongst our hospitals, but a more comprehensive scheme of organisation is essential, a scheme whereby our hospitals, instead of being conducted as detached and often incomplete units, shall be conducted as the units of one great Service, acting in unison, but otherwise not under control other than that of the local boards of managers.

This improvement in the efficiency of the Voluntary Service might be brought about by: (*a*) The establishment of district hospitals boards composed of representatives of boards of managers of groups of hospitals in areas to be defined, and vested with such limited powers as might be found necessary for the transaction of the business of the boards; each district board acting as a link between the hospitals of each group. (*b*) The establishment of a central hospitals board, composed of representatives of the district boards, to act as a link between the district boards.

The duties of the district boards would be: (1) To meet periodically and to discuss the needs of the areas represented, and mutually to arrange for the allocation of certain classes of cases to the best possible advantage. (2) To confer with the Central Board when co-operation between one district and another may be deemed desirable. (3) To define areas of population to be served by each hospital; to collect statistics from each hospital as to the localities from which the patients treated have been drawn, and to act as clearing houses for the transfer of funds from one hospital to another in respect of patients treated outside the areas defined for each hospital. (4) To discuss methods to be adopted for raising funds with a view to mutual help and, if possible, also with the object of effecting economy in the cost of collection.

It might be found desirable for the district boards to appeal for and collect funds by way of augmenting the individual efforts of each hospital; and if one may judge from the experience of the Metropolitan hospitals since the establishment of the King's Hospital Fund, this work might be undertaken without interfering to a material extent with the ordinary incomes of the hospitals. Such funds might be distributed amongst the hospitals of each group for the purposes of maintenance, or proportions might be reserved for the provision of special hospitals, extensions, convalescent homes, and the like. By the adoption of such a scheme as this the voluntary hospitals might, in time, be raised to that uniform standard of efficiency which the advocates of a State Service believe can be attained only by State control. For the Service would derive all the advantages of central control without the disadvantages of interference with local responsibility and the depreciation of local enthusiasm.

